# Rhizosphere microorganisms can influence the timing of plant flowering

**DOI:** 10.1186/s40168-018-0615-0

**Published:** 2018-12-26

**Authors:** Tao Lu, Mingjing Ke, Michel Lavoie, Yujian Jin, Xiaoji Fan, Zhenyan Zhang, Zhengwei Fu, Liwei Sun, Michael Gillings, Josep Peñuelas, Haifeng Qian, Yong-Guan Zhu

**Affiliations:** 1 College of Environment, Zhejiang University of Technology, Hangzhou, 310032 People’s Republic of China; 2 Quebec-Ocean and Takuvik Joint International Research Unit, Université Laval, Québec, G1VOA6 Canada; 3 College of Biotechnology and Bioengineering, Zhejiang University of Technology, Hangzhou, 310032 People’s Republic of China; 4 Department of Biological Sciences, Macquarie University, Sydney, NSW 2109 Australia; 5 CSIC, Global Ecology Unit, CREAF-CSIC-UAB, Barcelona, Catalonia Spain; 6 CREAF, Cerdanyola del Vallès, Barcelona, Catalonia Spain; 7 Xinjiang Key Laboratory of Environmental Pollution and Bioremediation, Xinjiang Institute of Ecology and Geography, Chinese Academy of Sciences, Urumqi, 830011 People’s Republic of China; 8 Key Lab of Urban Environment and Health, Institute of Urban Environment, Chinese Academy of Sciences, Xiamen, 361021 People’s Republic of China; 9 State Key Lab of Urban and Regional Ecology, Research Center for Ecoenvironmental Sciences, Chinese Academy of Sciences, Beijing, 100085 People’s Republic of China

**Keywords:** Rhizosphere, Microbiota, Root exudate, Nitrogen, Indole acetic acid, *Arabidopsis*, Flowering time

## Abstract

**Background:**

Plant phenology has crucial biological, physical, and chemical effects on the biosphere. Phenological drivers have largely been studied, but the role of plant microbiota, particularly rhizosphere microbiota, has not been considered.

**Results:**

We discovered that rhizosphere microbial communities could modulate the timing of flowering of *Arabidopsis thaliana*. Rhizosphere microorganisms that increased and prolonged N bioavailability by nitrification delayed flowering by converting tryptophan to the phytohormone indole acetic acid (IAA), thus downregulating genes that trigger flowering, and stimulating further plant growth. The addition of IAA to hydroponic cultures confirmed this metabolic network.

**Conclusions:**

We document a novel metabolic network in which soil microbiota influenced plant flowering time, thus shedding light on the key role of soil microbiota on plant functioning. This opens up multiple opportunities for application, from helping to mitigate some of the effects of climate change and environmental stress on plants (e.g. abnormal temperature variation, drought, salinity) to manipulating plant characteristics using microbial inocula to increase crop potential.

**Electronic supplementary material:**

The online version of this article (10.1186/s40168-018-0615-0) contains supplementary material, which is available to authorized users.

## Background

Climate change has altered plant phenology [1, 2]. This has crucial biological, physical, and chemical effects on the biosphere and the earth system [3]. These phenological alterations have become a subject of great interest in ecological and environmental sciences. Changes to phenology have been attributed to multiple factors, including warming [4], but the role of plant microbiota and particularly rhizosphere microbiota has not been considered. And yet, the rhizosphere harbors a diverse community of microorganisms that play critical roles in plant growth and reproduction [5, 6].

We know that rhizosphere microbiota protect against pathogens, improve growth by producing phytohormones, and may help plants withstand environmental perturbations such as abnormal variation in temperature, drought, and salinity related to climate [7–10]. Recent studies also suggest that root microbiota can contribute to phenotypic plasticity, which has important implications for our understanding of plant phenology in a changing climate and for increasing crop production [11, 12]. Several auxins have decisive functions in the establishment of plant developmental and reproductive programs [13, 14]. Auxins can be synthesized by rhizosphere microorganisms [15, 16], raising the intriguing possibility that root microbiota may regulate plant growth and development through phytohormone production.

Understanding the interactions between plant microbiota, root exudation, and plant growth and reproduction, however, remains limited, despite significant advances in the last decade [17]. Root exudates account for 5–21% of total photosynthetically fixed carbon and help drive the composition of rhizosphere communities [18, 19]. Exudates may be excess plant products [20, 21], but they can also contain signaling and chemoattractant molecules. These molecules recruit beneficial microorganisms that contribute to pathogen resistance, water retention, and the synthesis of growth-promoting hormones [22], and may influence plant phenotype [23].

Interactions between exudates, soil microbiota, and plant physiology have the potential to dynamically affect rhizospheric communities and alter plant phenotypes by complex feedback mechanisms. We studied the molecular interactions among root exudates, rhizosphere microbiota, and plant physiology in wild-type (Wt) and mutant (*pgr*5) plants of *Arabidopsis thaliana* (hereafter *Arabidopsis*) and identified a novel network of molecular interactions linking the nitrogen cycle, the phytohormone IAA produced from Tryptophan (Trp), and the timing of flowering. These results thus provide evidence of an outstanding phenomenon: the timing of plant flowering may be influenced by soil microbiota.

## Results and discussion

### Rhizosphere microbiota can delay the onset of flowering of Wt *Arabidopsis*

**Fig. 1:**
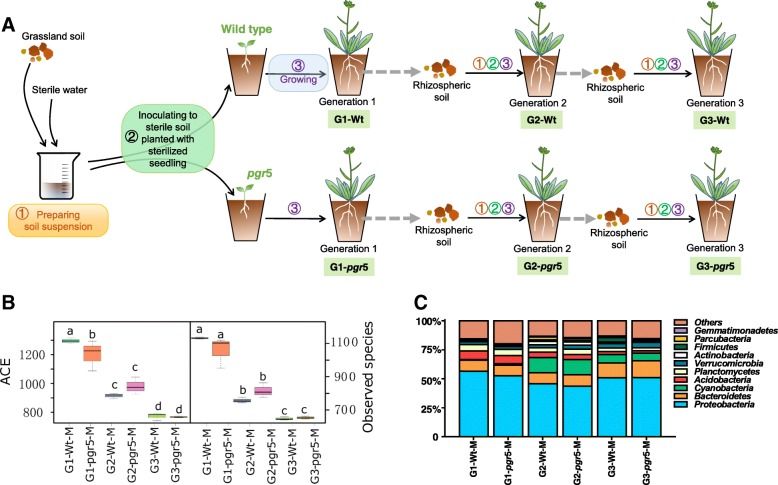
Design of the microcosmic experiment across generations and the profiles of rhizosphere microbial communities. **a** The experimental operation diagram of soil microbiota selection by plants; rhizosphere microbial richness: ACE index and number of species per sample (**b**); and relative abundance of the 10 most abundant microbial phyla (**c**) across three generations of *Arabidopsis* grown in microcosms. G-Wt-M represents microbiota in the corresponding generation of wild-type; G-*pgr*5-M represents microbiota in the corresponding generation of *pgr*5 *Arabidopsis*. Taxonomy details and significance analysis are shown in Additional file 2. Different letters represent significant differences (ANOVA followed by an LSD test; *p* < 0.05). Values are means ± SDs (*n* = 3)

Multiple generations of experimental adaptation/acclimation could be used to observe microbially mediated mechanisms of plant growth and reproduction [24–26]. To test whether the multi-generations of rhizosphere microbiota can induce earlier or delay flowering time, we measured the phenotypic parameters of *Arabidopsis* plants growing in soil for three generations (G1, G2, or G3) inoculated with different soil microbiomes (Fig. 1a). The phenotypic parameters of Wt *Arabidopsis* did not change significantly in plants treated with microbiota isolated from the roots of wild-type plants (Wt-M treatment) compared to the corresponding control (growing in sterilized soil without addition of soil microbiomes) during the first two generations (Additional file 1: Table S1). This indicates that plants in sterilized soil could grow as well as those grown in sterilized soil inoculated with a live microbial slurry during the first two generations. Wt-M rhizosphere microbiota significantly affected flowering and reproduction by the third generation. Flowering time in the G3-Wt-M group was significantly delayed, by approximately 3 days, and silique number increased significantly.

The richness and diversity of the rhizosphere microorganisms tended to decrease in parallel with the changes in *Arabidopsis* physiology over the three generations. Species richness and diversity based on four indices (Chao1, abundance-based coverage (ACE), number of species, and the Shannon index) significantly decreased after the two and three generations (Fig. 1b and Additional file 1: Figure S1). The relative abundances of *Proteobacteria* and *Acidobacteria*, the dominant bacterial phyla in the rhizosphere, decreased by generations 2 and 3 relative to generation 1. In contrast, *Bacteroidetes* and cyanobacterial abundances increased by generation 3 (Fig. 1c and Additional file 2). It was demonstrated that some early (or late)-flowering plant could be associated to specific soil microorganism families [25] and that plant microbiota could be associated with changes in different plant growth phases [27]. The results suggest that the selected enriched microbes during the three generations play crucial roles in modulating plant flowering time.

### Rhizosphere microbiota advanced the flowering time of the *pgr*5 mutant

To better understand the relationships between microbiota, exudates, and flowering time, we used an *Arabidopsis* mutant (for the PGR5 gene that encodes a novel thylakoid membrane protein) that grows as well as Wt plants in the vegetative phase [28, 29]. The *pgr*5 mutant is deficient in antimycin A-sensitive cyclic flow from ferredoxin to plastoquinone, which is one of the most crucial physiological processes for efficient photosynthesis [28]. Because of defects in photosynthesis, the *pgr*5 mutant produces different exudates from the Wt *Arabidopsis*. The phenotype of the *pgr*5 mutant was unchanged in generations 1 and 2 in the group treated with *pgr5* microbiota (*pgr*5-M) compared to the corresponding control (without the addition of soil microbiomes), as for the Wt *Arabidopsis*. The flowering time of the *pgr*5-M-treated group, however, was nearly 4 days earlier by generation 3, in contrast to Wt, and the silique number was significantly lower (Additional file 1: Table S1). The changes in plant reproduction induced by the microbial manipulations were probably not driven by rhizosphere diversity, because the Shannon and richness indices of the *pgr*5-M- and Wt-M-treated groups did not generally differ over the three generations (Additional file 1: Figure S1). The ACE data, though, differed after one generation (Fig. 1b). The relative abundances of the phyla differed only marginally between the two *Arabidopsis* lines in each generation (Fig. 1c).

### Rare rhizosphere microbes potentially affected flowering time

The abundances of microbial phyla were relatively constant (see above), but abundances between the WM- (microbiota from the third-generation Wt cultures) and the PM (microbiota from the third-generation *pgr*5 cultures)-treated groups differed more at lower taxonomic levels. The relative abundances of 77 rhizosphere genera differed significantly between the WM and PM treatment by generation 3, by at least a factor of two. A total of 41 genera were enriched in the WM treatment relative to the PM treatment, and 36 genera were enriched in the PM treatment (Additional file 1: Table S2 and Additional file 3).

Most of the enriched rhizosphere microorganisms were initially rare (relative abundance < 1%), such as *Emticicia*, *Methylobacterium*, and *Filimonas*, suggesting that rare rhizosphere microbes might play a role in modulating *Arabidopsis* flowering. Rare microbes can be involved in soil biochemical processes and as active modulators of plant growth and resistance to pathogens [30]. The enriched microbes in the WM treatment mostly have key roles in rhizosphere N regeneration or in maintaining plant growth (Additional file 1: Table S3) [16, 31–34]. Indeed, *Bacillus*, enriched in the WM treatment, can potentially contribute to soil N fixation [35]. Potential denitrifying organisms such as *Stenotrophomonas* and *Emticicia*, though, were enriched in the PM treatment [31, 36]. We hypothesize that an increase in N fixation and cycling in Wt *Arabidopsis* associated with the microbiota, and the potential increase in denitrification in the *pgr*5 mutant, could help increase the duration of N bioavailability in Wt *Arabidopsis* relative to the *pgr*5 mutant. Plant pathogenic genera enriched in PM, such as *Panacagrimonas* and *Filimonas*, may also contribute to the earlier flowering time, because infected hosts preferentially allocate resources toward reproduction [37, 38]. This hypothesis linking N availability to flowering time is discussed further below within the newly proposed molecular network that modulates flowering time.

### Verification of microbial function

**Fig. 2:**
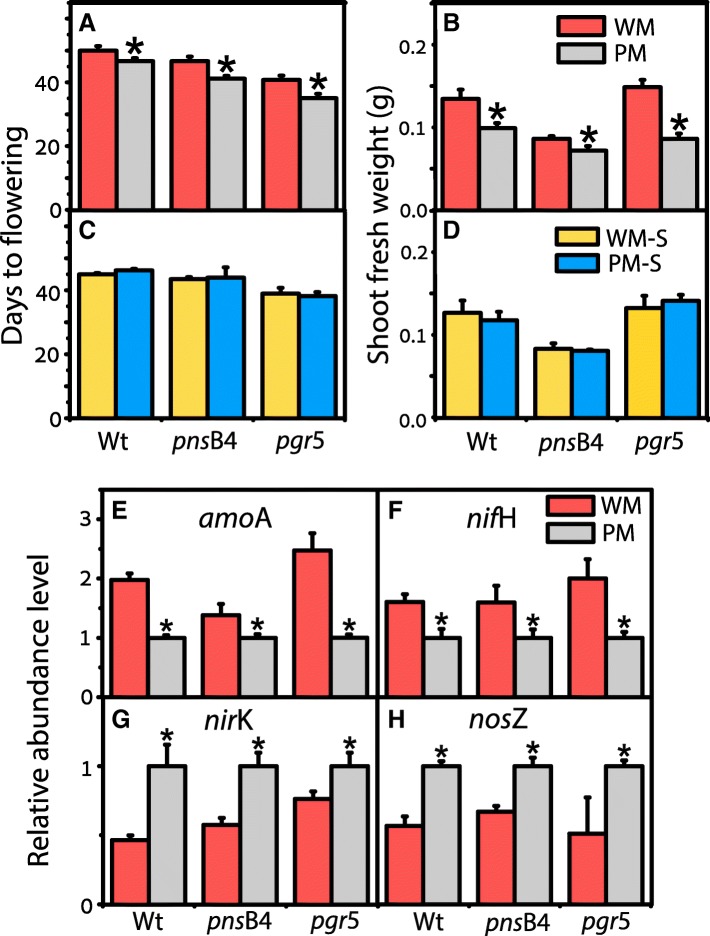
Verification of microbial function from the third generation. Number of days to the onset of flowering (i.e., when 80% of the control plants had floral buds of 1 cm or larger) (**a**, **c**) and shoot fresh weight (**b**, **d**) in wild-type (Wt) and two mutants (*pns*B4 and *pgr*5) of *Arabidopsis* grown in microcosms in the presence of unsterilized soil slurry (WM and PM) (**a**, **b**) or sterilized soil slurry (WM-S and PM-S) (**c**, **d**) from the third generation. WM represents plants with microbiota isolates from wild-type plants in generation 3, PM represents the plants with microbiota isolates from *pgr*5 plants in generation 3, WM-S represents wild-type *Arabidopsis* grown with sterilized soil slurry, and PM-S represents *Arabidopsis pgr*5 mutants grown with sterilized soil slurry. Relative abundance of genes involved in N cycling (**e** *amo*A, ammonia oxidation; **f** *nif*H, N fixation; **g** *nir*K, nitrite reductase; **h** *nos*Z, nitrous oxide reductase) in soil samples of Wt and two mutant (*pns*B4 and *pgr*5) lines of *Arabidopsis* grown in microcosms with the addition of unsterilized rhizosphere soil slurries of wild-type (WM) and mutant (PM) *Arabidopsis* cultures. Asterisk represents a significant difference between WM and PM (*p* < 0.05). Values are means ± SDs (*n* = 9 with 20 *Arabidopsis* plants per sample)

We unambiguously demonstrated that flowering time was directly associated with the rhizosphere microbiota. Microbiota from the third-generation *pgr*5 (PM) or Wt cultures (WM) was used to inoculate cultures of three *Arabidopsis* lines (Wt and two mutants of the photosynthetic apparatus, *pgr*5 and *pns*B4, deficient in cyclic electron flow from NADPH to plastoquinone) for one generation. The microbiota of the third-generation Wt culture delayed flowering time and increased shoot growth in all cases compared to the treatment using microbiota from the *pgr*5 cultures. The addition of Wt microbiota delayed flowering by 3.3, 5.5, and 5.7 days in Wt, the *pns*B4 mutant, and the *pgr*5 mutant, respectively (Fig. 2a). The shoot fresh weight of the plants treated with Wt microbiota also increased significantly in the three lines compared to the treatments with the addition of *pgr*5 microbiota (Fig. 2b).

**Fig. 3:**
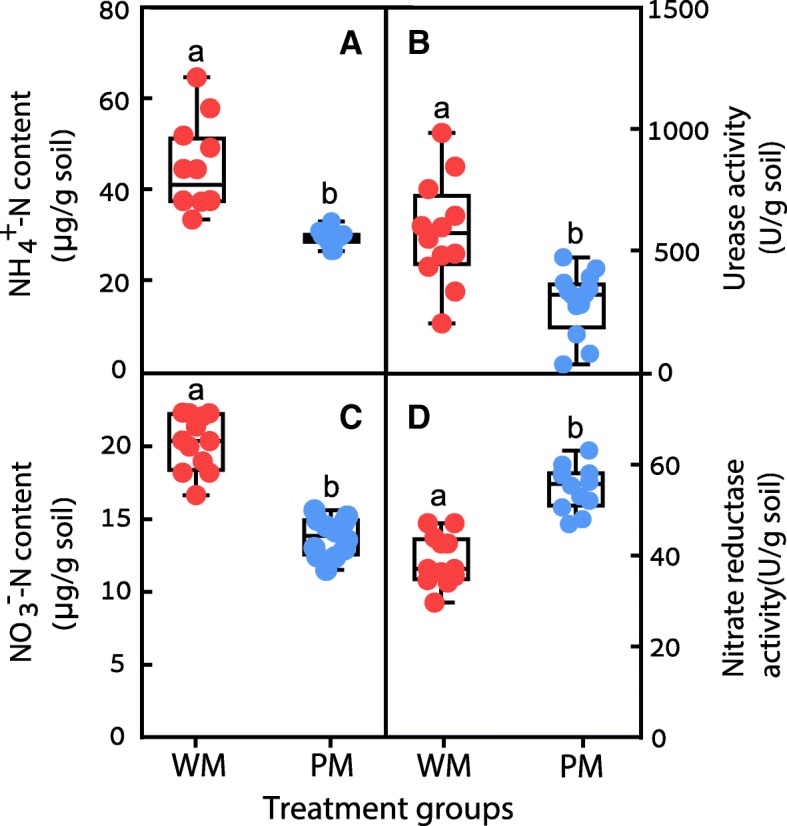
N availability could be influenced by the addition of microbiota from the third generation. NH_4_^+^-N content (**A**), urease activity (**B**), NO_3_^−^-N content (**C**), and nitrate reductase activity (**D**) after inoculation for the WM- and PM-treated groups in the three *Arabidopsis* lines (Wt, *pns*B4, and *pgr*5) (*n* = 12). Different letters represent significant differences (*p* < 0.05). The red symbols represent the WM-treated samples, and the blue symbols represent the PM-treated samples

These results clearly indicate that flowering time can be affected by the rhizosphere microbiota. This effect disappeared when the soil slurry was sterilized before inoculation, indicating that heat-stable exudates alone were not modulating *Arabidopsis* flowering time (Fig. 2c, d). It is interesting that the difference between WM- and WM-S (sterilized soil slurry)-treated plants was not very obvious (Additional file 1: Figure S4). The reason may be that sterilized soil slurry included more N, and other nutrients to influence flowering time, which was in accordance with the results in Fig. 3. Therefore, we speculated that the metabolites in the sterilized slurry also played a role in influencing the flowering time and plant stature.

The addition of the two soil slurries did not change bulk-soil pH, available soil K or P contents (Additional file 1: Table S4), the abundance of key genes involved in the C cycle, or the activities of β-glucosidase or chitinase in the rhizosphere (Additional file 1: Figure S2 and S3) but did affect the abundance of genes (normalized to the abundance of 16S rRNA gene) involved in N cycling (Fig. 2e–h) and the amounts of NH_4_^+^ (Fig. 3A) and NO_3_^−^ (Fig. 3C) in soil. The concentration of bioavailable N species was generally lower after the addition of the *pgr*5 microbiota compared to the treatment with the addition of Wt microbiota (Fig. 3A, C). This decrease in N availability in the cultures treated with *pgr*5 microbiota was accompanied by an increase in the abundance of genes involved in denitrification (*nir*K and *nos*Z) and a decrease in the abundance of genes involved in nitrogen fixation (*nif*H) and nitrification (*amo*A) compared to the Wt-treated groups (Fig. 2e–h). In the Wt-treated group, the activity of urease was higher (Fig. 3B) and nitrate reductase was lower (Fig. 3D) than those in the PM group, which probably resulted in higher NH_4_^+^ and NO_3_^−^.

Several lines of evidence support the hypothesis that the rhizosphere microbiota modulated N cycling and bioavailability, leading to N deficiency earlier in the *pgr5*-treated groups and thus earlier flowering. Flowering can be triggered by low nitrate levels [39], but plants maximize growth before flowering under the conditions of N sufficiency [25].

### Different root exudates in the two *Arabidopsis* lines

**Fig. 4:**
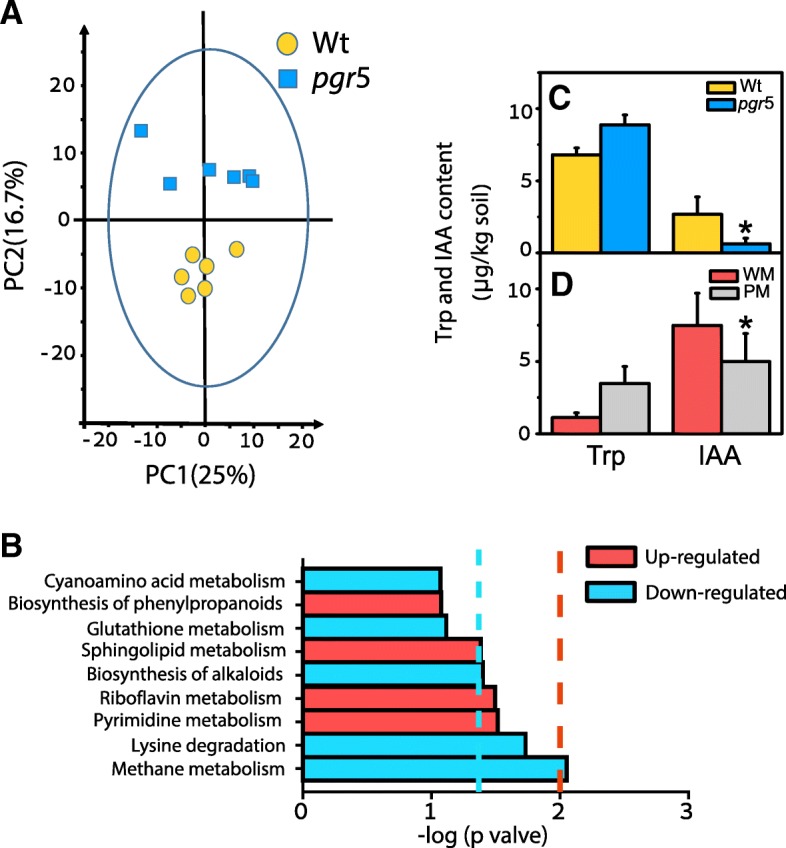
Root exudates of the two *Arabidopsis* lines. **a** Principal component analysis (PCA) of root exudates in the Wt and *pgr*5 treatments (*n* = 6) grown hydroponically. **b** Differentially released exudates (*p* < 0.05) in the Wt and *pgr*5 treatments were classified into corresponding metabolic pathways inferred from the KEGG pathway database (*n* = 6). **c** Tryptophan (Trp) and indole-3-acetic acid (IAA) contents in the soil of the cultures of Wt *Arabidopsis* or *pgr5* mutants grown for three generations in microcosms. **d** Trp and IAA contents in the soil-cultured Wt *Arabidopsis* for one generation that had been in advance added the microbiota from the Wt (WM) or the *pgr*5 mutant (PM) *Arabidopsis*. Red and blue bars indicate that the exudates for a given pathway are mainly up- or downregulated, respectively, in the Wt cultures relative to the *pgr*5 mutant cultures. The blue and red vertical dashed lines depict *p* = 0.05 (−log (0.05) = 1.3) and *p* = 0.01 (−log (0.01) = 2), respectively. Asterisk represents a significant difference (*p* < 0.05). Values are means ± SDs (*n* = 6)

Root exudates can act as key substrates or signaling molecules that affect microbial composition [27], so we tested the hypothesis that exudate concentrations and compositions differed between the *Arabidopsis* lines. A metabolomic analysis found that 34 exudates involved in 10 metabolic pathways were differentially released in the two lines (Wt and *pgr*5) (see Additional file 1: Table S5, the principal component analysis in Fig. 4a, and ≥ 2- or ≤ 0.5-fold changes and *p* values < 0.05 in Fig. 4b). Four of the 10 biochemical pathways were upregulated in the Wt cultures relative to the *pgr*5 cultures (Fig. 4b). Thymine was the most differentially released exudate (Additional file 1: Table S5). Thymine can be degraded by bacteria, perhaps accounting for the increase in NH_4_^+^ content in the WM groups (Fig. 3A).

Trp and its derivatives, phenols, and some carboxylic acids were preferentially exuded in the Wt cultures (Additional file 1: Table S5). The concentrations of amino acids were generally higher in the Wt cultures, consistent with a higher abundance of *Bacillus subtilis* in the Wt cultures, for which amino acids are chemoattractants. These metabolomic results are consistent with the hypothesis that differences in exudates affect rhizosphere microbiota [40].

### IAA delayed flowering time by downregulating genes involved in flowering

Auxins regulate plant growth in many ways [13, 41, 42]. One of the important auxins for plants is IAA, which is soluble in aqueous solutions and, when protonated, diffuses passively across cell membranes without the need of a specific transporter [43]. IAA has also been hypothesized to have a floral-inductive signaling role by regulating multiple aspects of embryonic and postembryonic development [44, 45]. Microorganisms can produce IAA from Trp [46–49]. One of the enriched rhizosphere microorganisms, *Arthrobacter* (Additional file 1: Table S3), has been reported to be beneficial for plant growth by having the ability to produce IAA [16]. Trp and its derivatives were enriched in the Wt exudates, so the generation of IAA by microorganisms may control flowering time by a novel molecular network. Trp content in the soil of generation 3 of the Wt cultures decreased, and the IAA content increased 3.03-fold, suggesting that the Wt microbiota rapidly converted Trp into IAA (Fig. 4c, d).

**Fig. 5:**
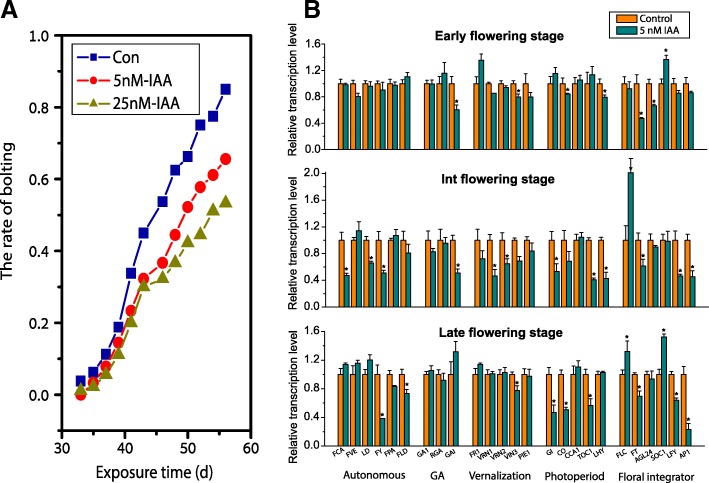
IAA delayed flowering time by downregulating genes involved in flowering. **a** Proportion of bolting (i.e., percentage of plants had floral buds of 1 cm or larger). Plants were hydroponically grown in MS medium containing 5 nM and 25 nM IAA. **b** Relative rates of transcription of genes involved in flowering in Wt *Arabidopsis* at the early, intermediate (Int.), and late stages of flowering after one generation of culture. Plants were hydroponically grown in MS medium containing 5 nM IAA. All transcription levels are normalized to that of a housekeeping gene (actin 2). Values are means ± SDs (*n* = 6). Asterisk represents a significant difference (*p* < 0.05)

We explored the possibility that microbially generated IAA delayed flowering time by adding Trp, 5-hydroxytryptophan (5-HTP), and IAA to the hydroponic cultures and monitoring the flowering time of each line. Adding 5 and 25 nM IAA decreased the bolting proportion of Wt *Arabidopsis* by 20–30% (Fig. 5a) when 80% of the control plants had floral buds, implying that IAA was the direct driver that delayed flowering time. Changes in the expression of genes involved in flowering further supported the role of IAA in regulating flowering time (Fig. 5b). IAA treatment induced changes in the relative transcription rates of genes associated with flowering. The rates for some of the genes comprising the autonomous, GA, and vernalization pathways changed significantly, and these changes were observable at the early (when 10% of control plants had floral buds), intermediate (when > 50% of control plants had floral buds), and late (when > 80% of control plants had floral buds) stages of flowering. The downregulation of gene activity was most consistent in the photoperiod pathway, where multiple genes were downregulated at all flowering stages.

Shimada et al. [14] reported that IAA relieved the inhibitory effects of aspterric acid on pollen growth and thus speculated that IAA accelerated reproductive growth in *Arabidopsis*. To our knowledge, our microcosm study is the first to demonstrate that IAA is one critical signal that delays flowering in *Arabidopsis*, although Wagner et al. [50] also found a similar phenomenon, while not proposing any related mechanism.

Combined with earlier reports, the weight of evidence suggests that IAA stimulates the development of floral organs but delays the flowering time of *Arabidopsis*. Our data strongly suggest that IAA delays flowering and acts as a signal of optimal growth conditions in the absence of N limitation for growth.

## Conclusions

**Fig. 6:**
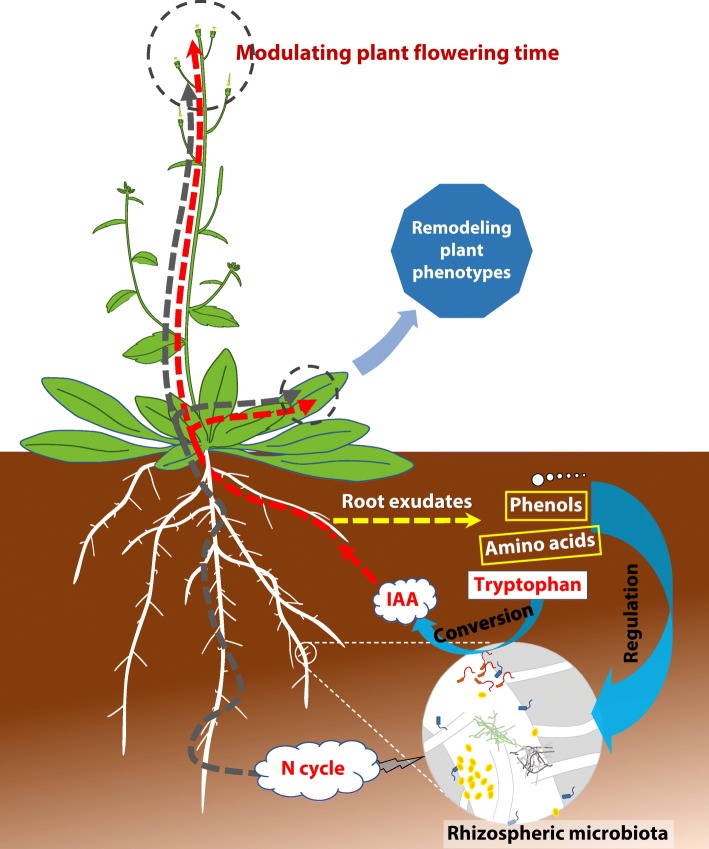
Schematics of interactions between plants and rhizosphere microbiota. The proposed community/function relationships for rhizosphere microorganisms and their interaction with plant physiology and floral phenology through root exudates, N cycling, and production of the phytohormone IAA

Identifying the community/function relationships for rhizosphere microorganisms and their interaction with plant physiology is critical for determining the role of the plant microbiome in regulating biogeochemical cycles, plant growth, and phenology (Fig. 6). We identified a novel metabolic network in which exudates affect plant rhizosphere microbiota, which can then modulate flowering time by IAA production and can also affect vegetative growth by influencing N availability. IAA-promoted plant growth is expected to further stimulate exudation and hence retroactively affect flowering time in a positive feedback mechanism. Our results have important implications for our understanding and modeling of plant phenology and are of great interest for the biotechnology sector seeking to increase crop potential.

## Methods

### Seedling culture

*Arabidopsis* seeds (wild-type Col-0 (Wt) and the *pgr*5 mutant, deficient in *PGR*5-dependent cyclic electron flow from ferredoxin to plastoquinone), were surface sterilized to avoid bacterial contamination on solid medium and vernalized as described by Sun et al. [51]. Given that photosynthesis is the main resource of exudates, photosystem mutant with the same genetic background with that of Wt was selected to induce different exudates. Vernalized seeds were cultured in Petri dishes containing Murashige and Skoog (MS) medium under sterile conditions at 25 °C at a light intensity of 300 μmol photons/m^2^/s and a 12:12-h light to dark photoperiod. The MS medium containing 3% sugar and 0.5% agar was autoclaved at 115 °C for 30 min before use. Two-week-old aseptic seedlings were transplanted into polycarbonate pots (400 mL) containing autoclaved potting-mix soil (Sun Gro Horticulture, MA, USA).

### Microcosm experiments across generations

Approximately 30 g of grassland soil collected near the Zhejiang University of Technology, China (30° 17′ 45.11″ N, 120° 09′ 50.07″ E) was mixed with 200 mL of sterile water by vigorous shaking for 60 s [25]. Ten-milliliter samples of soil slurry were added to polycarbonate pots transplanted with 20 seedlings of either the Wt or the *pgr*5 *Arabidopsis* line. Nine replicate pots were used for generation 1 for each line and for a control treatment (*n* = 9) without added soil slurry. Plants grew in an artificial greenhouse at 25 ± 0.5 °C and 80% relative humidity under cool-white fluorescent light (300 μmol photons/m^2^/s) with a 12:12-h light to dark cycle.

The plants and soils were harvested when 80% of the plants had floral buds of 1 ± 0.1 cm or larger (measured from the center of the rosette), and the time to flowering was recorded. Large soil aggregates that were loosely bound to the roots were first removed by shaking, and 30 g of the tightly bound rhizosphere soil was then collected (see the “Collection of rhizosphere soil” section) and added to 200 mL of sterile water, producing a soil slurry for generation 1. This slurry was used for inoculating the *Arabidopsis* lines for the second and third generations, as described above. Treatments inoculated with the microbiota of Wt and the *pgr*5 mutant were designated WM and PM, respectively. The generations in our study refer to the propagation of soil microbes only, and all seeds were from a common stock. At flowering stage and harvest time, the physiological parameters including the number of days before flowering, fresh weight, number of rosette leaves, and number of siliques were determined in wild-type (Wt) and pgr5 mutant ecotypes grown in microcosms for each generation.

### Collection of rhizosphere soil

Rhizosphere soil was collected as described by Bulgarelli et al. [9]. Four-centimeter sections of roots were collected immediately below the rosette, and the roots from each pot were transferred to a 50-mL centrifuge tube containing 20 mL of sterile phosphate buffer saline (PBS; 137 mM sodium chloride, 10 mM phosphate buffer, 2.7 mM potassium chloride; pH 7.3–7.5). The centrifuge tubes were shaken at 40 g for 20 min on an orbital shaker. The roots were removed, the solution was centrifuged at 1000*g* for 20 min, and the pellet of rhizosphere soil was recovered. All samples of rhizosphere soil were frozen in liquid nitrogen and stored at − 80 °C until analysis.

### 16S rRNA gene sequencing

The frozen soil samples were thawed on ice, and DNA was extracted using a PowerSoil DNA Isolation Kit (MO BIO Laboratories, Inc., Carlsbad, USA). 16S rRNA genes were amplified using EXtaq enzyme (TaKaRa, Kyoto, Japan) and the specific primers 314F (5′-CCTACGGGNGGCWGCAG-3′) and 805R (5′-GACTACHVGGGTATCTAATCC-3′) with the adapter (index) that targets the V3 and V4 variable regions of bacterial/archaeal 16S rRNA genes. Strongly amplified products 460 bp in length were chosen for further experiments. The amplicons were quantified with a Qubit 2.0 fluorometer (Thermo Fisher Scientific, USA), diluted to 1 ng/μL, and sequenced on a MiSeq platform (PE250). In total, 854,514 raw reads were obtained from the 18 samples of rhizosphere soils (6 groups as shown in Fig. 1a, *n* = 3). We computed operational taxonomic units (OTU) and microbial diversity as described previously [52]. Rarefaction curves of observed species are shown in Additional file 1: Fig. S5.

### Analysis of rhizosphere DNA abundance by quantitative real-time PCR (qRT-PCR)

Rhizosphere DNA was quantified by ultraviolet-visible spectrophotometry (ND 5000, BioTeke, China) and diluted to 10 ng/μL. The abundances of rhizosphere genes (*amo*A, *nif*H, *nir*K, *nos*Z, *cbb*L, β-glu, and *Chi*A; Additional file 1: Table S6) were measured in 10-μL PCRs containing 5 μL of SYBR Green Real-time PCR Master Mix (Toyobo, Tokyo, Japan), 0.4 μL of each of the forward and reverse primers (10 mM) (see Additional file 1: Table S6 for a list of primers), 1 μL of rhizosphere DNA at 10 ng/L, and 3.2 μL of ddH_2_O. The qRT-PCR protocol was 94 °C for 3 min followed by 40 cycles of 94 °C for 30 s, 56 °C for 30 s, and 72 °C for 1 min on an Eppendorf MasterCycler ep RealPlex (Wesseling-Berzdorf, Germany).

### Measurements in the dissolved phase of the soil samples

Physicochemical (nutrient contents and pH) and biological (enzymatic activities and concentrations of IAA and Trp) variables were measured in the dissolved phase of the soil samples. After three generations of *Arabidopsis* cultures (Fig. 1a), soil samples were then collected randomly from each pot, air-dried, homogenized, and sieved to obtain particles < 1 mm. The activity of soil enzymes (β-glucosidase, chitinase, urease, and nitrate reductase) and soil N content (NH_4_^+^ and NO_3_^−^ contents) were subsequently measured using 0.5 g of soil (*n* = 4) following the manufacturer’s instructions of corresponding commercial reagent kits (COMIN, Suzhou, China). The concentrations of two key metabolites (IAA and Trp) were measured in the soil dissolved phase. Five grams of soil was mixed with 5 g of NaCl and 20 mL of acetonitrile for 5 min, followed by centrifugation at 1000*g* for 10 min. The concentrations of IAA and Trp in the supernatants were measured by Liquid Chromatography-Electrospray Ionization-Mass/Mass Spectroscopy (LC-ESI-MS/MS, Q-Trap 5500, Agilent Technologies, USA).

### Collection, measurement, and identification of root exudates

*Arabidopsis* seedlings (Wt and *pgr*5 mutant) reached the bolting stage after approximately 40 days of culture and were then transferred to glass containers containing 40 mL of ddH_2_O [27]. After 3 days of culture in the glass containers, culture media containing exudates were passed through 0.45-μm filter membranes. Approximately 35 mL of the solution was freeze-dried, dissolved in 500 μL of 80% methanol, and derivatized with Bis (trimethylsilyl) trifluoroacetamide and 1% chlorotrimethylsilane. The derivatized samples were analyzed by gas chromatography/mass spectroscopy GC-MS (Agilent 7890B gas chromatographic system coupled to an Agilent 5977A Mass Spectrometry Detector (Agilent, USA)). The potential involvement of all identified exudates in *Arabidopsis* biochemical pathways was subsequently determined by reference to the KEGG database [52].

### Addition of Trp, 5-hydroxytryptophan (5-HTP), and IAA and analysis of gene transcription associated with flowering

For hydroponic experiments (i.e., plants cultured in solution), Trp, 5-HTP, and IAA were added to autoclaved liquid MS medium cultured with Wt at final concentrations of 0.5, 5, and 25 nM, respectively. Control cultures without Trp, 5-HTP, and IAA were performed in parallel. The transcription of genes involved in flowering was measured by qRT-PCR. Control groups treated with Trp, 5-HTP, and IAA were analyzed at three stages of development: (1) early flowering (when 10% of control plants had floral buds), (2) intermediate flowering (when > 50% of control plants had floral buds), and (3) late flowering (when > 80% of control plants had floral buds) stages. Total RNA was isolated from the plants and then reverse transcribed into cDNA for qRT-PCR analysis using the protocol described by Chen et al. [11] and the primer pairs in Additional file 1: Table S6. We studied the transcription of six genes in the autonomous pathway (FCA, FLD, FPA, FVE, FY, and LD), three genes in the gibberellin acid (GA) pathway (GAI, GA1, and RGA), six genes in the vernalization pathway (FR1, VRN1, VRN2, VIN3, and PIE1), five genes in the photoperiod pathway (CCA1, CO, GI, LHY, and TOC1), and five genes in the floral integrator pathway (FLC, FT, SOC1, AGL-24, LFY, and AP1).

### Data analysis and statistical methods

For the biochemical and physiological measurements, analysis of variance followed by the Dunnett’s post hoc test was performed to evaluate the statistical significance among data using the StatView 5.0 program (Statistical Analysis Systems Institute, Cary, NC, USA). Means among treatments were considered significantly different, when the probability (*p*) was less than 0.05. All analyses were performed in triplicate unless otherwise stated. All data are presented in the tables and figures as mean ± SD (standard deviation).

## Additional files

Additional file 1: **Figure S1.** Rhizosphere microbiota richness and diversity. **Figure S2.** Abundance of carbon cycle-related genes in rhizosphere soil. **Figure S3.** Activities of carbon cycle-related enzymes in rhizosphere soil. **Figure S4.** Comparisons between WM- and WM-S-treated plants. **Figure S5.** Rarefaction curves of observed species. **Table S1.** Physiological parameters of *Arabidopsis* in three generations. **Table S2.** Significant enrichment of rhizosphere microorganisms in the third generation. **Table S3.** nriched rare microorganisms in Wt and *pgr*5 *Arabidopsis*. **Table S4.** Bulk soil properties measured after addition of WM and PM soil slurries. **Table S5.** Comparison of root exudates between Wt and *pgr*5 mutant *Arabidopsis*. **Table S6.** Sequences of the primer pairs used for qRT-PCR. (DOCX 348 kb)

Additional file 2: Taxonomy (relative abundance) of rhizosphere microorganisms at the phylum level. (XLSX 35 kb)

Additional file 3: Taxonomy (relative abundance) of rhizosphere microorganisms at the genus level. (XLSX 46 kb)
