# Supplementary material for: Rhizosphere microorganisms can influence the timing of plant flowering
Source: Microbiome. 2018 Dec 26;6:231. doi: 10.1186/s40168-018-0615-0 (PMC6307273; doi:10.1186/s40168-018-0615-0)
Supplement: Supplementary file 1 — Figure S1. Rhizosphere microbiota richness and diversity. Figure S2. Abundance of carbon cycle-related genes in rhizosphere soil. Figure S3. Activities of carbon cycle-related enzymes in rhizosphere soil. Figure S4. Comparisons between WM- and WM-S-treated plants. Figure S5. Rarefaction curves of observed species. Table S1. Physiological parameters of Arabidopsis in three generations. Table S2. Significant enrichment of rhizosphere microorganisms in the third generation. Table S3. nriched rare microorganisms in Wt and pgr5 Arabidopsis. Table S4. Bulk soil properties measured after addition of WM and PM soil slurries. Table S5. Comparison of root exudates between Wt and pgr5 mutant Arabidopsis. Table S6. Sequences of the primer pairs used for qRT-PCR. (DOCX 348 kb) [file 40168_2018_615_MOESM1_ESM.docx]

Additional File 1 for

**Rhizospheric microorganisms can influence the timing of plant flowering**

Tao Lu, Mingjing Ke, Michel Lavoie, Yujian Jin, Xiaoji Fan, Zhenyan Zhang, Zhengwei Fu, Liwei Sun, Michael Gillings, Josep Peñuelas, Haifeng Qian^*^, Yong-Guan Zhu^*^

^*^Correspondence authors. Email: hfqian@zjut.edu.cn (H.F.Q.), ygzhu@iue.ac.cn (Y.G.Z.)

**This file includes:**

Fig. S1. Rhizosphere microbiota richness and diversity.

Fig. S2. Abundance of carbon cycle-related genes in rhizosphere soil.

Fig. S3. Activities of carbon cycle-related enzymes in rhizosphere soil.

Fig. S4. Comparisons between WM and WM-S treated plants.

Fig. S5 Rarefaction curves of observed species.

Table S1. Physiological parameters of *Arabidopsis* in three generations.

Table S2. Significant enrichment of rhizosphere microorganisms in the third-generation.

Table S3. Enriched rare microorganisms in Wt and *pgr*5 *Arabidopsis*.

Table S4. Bulk soil properties measured after addition of WM and PM soil slurries.

Table S5. Comparison of root exudates between Wt and *pgr*5 mutant *Arabidopsis*.

Table S6. Sequences of the primer pairs used for qRT-PCR.


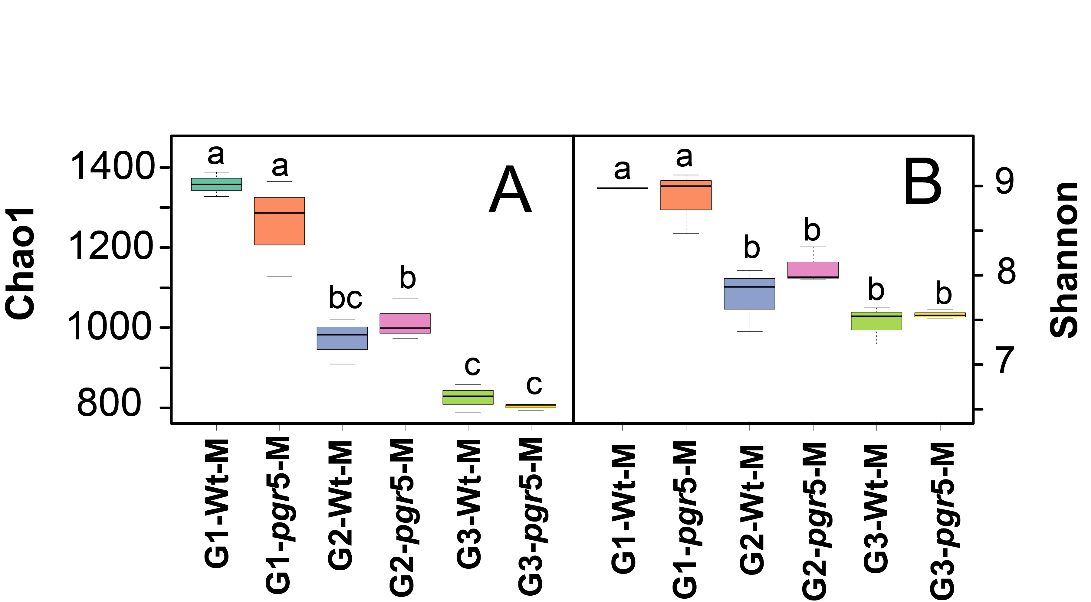


**Fig. S1. Rhizosphere microbiota richness and diversity.** Rhizosphere microbiota richness (Panel A: Chao1 index) or diversity (Panel B: Shannon index) across three generations of *Arabidopsis* grown in microcosms. Different letters represent significant differences (ANOVA followed by LSD test; *p*< 0.05). Values are mean ± SD (n = 3).


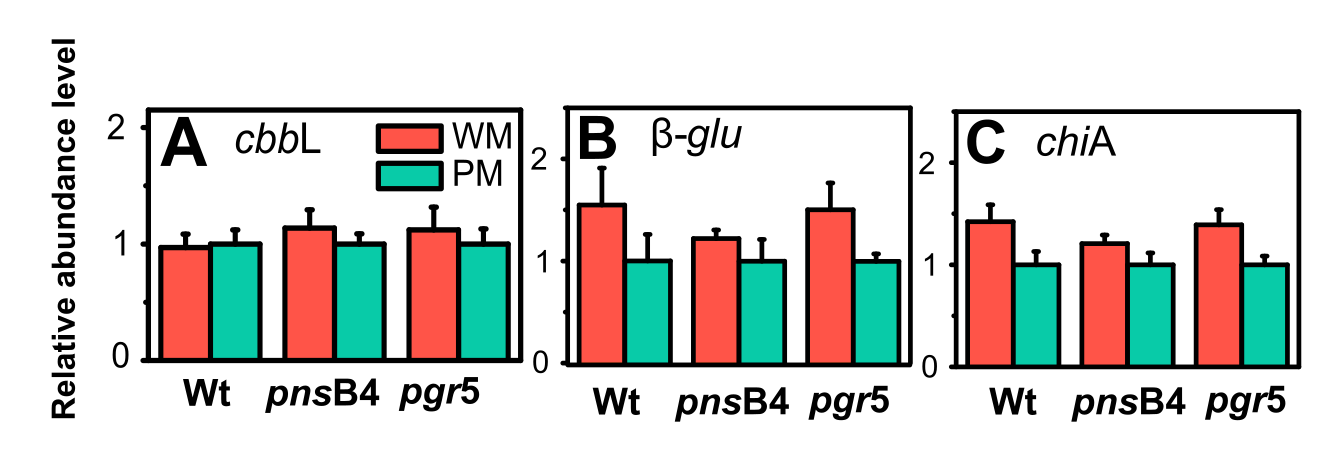


**Fig. S2. Abundance of carbon cycle-related genes in rhizosphere soil.** *cbb*L or ribulose 1, 5-bisphosphate carboxylase large subunit (**A**), β-*glu* or β-Glucosidase (**B**) and *chi*A or chitinase A **(C**) in rhizosphere soil of the wild-type (Wt) and two mutants (*pns*B4, *pgr*5) of *Arabidopsis* grown in microcosms in the presence of non-sterilized soil slurry (WM and PM) from three generations. Values are mean ± SD (n= 4).


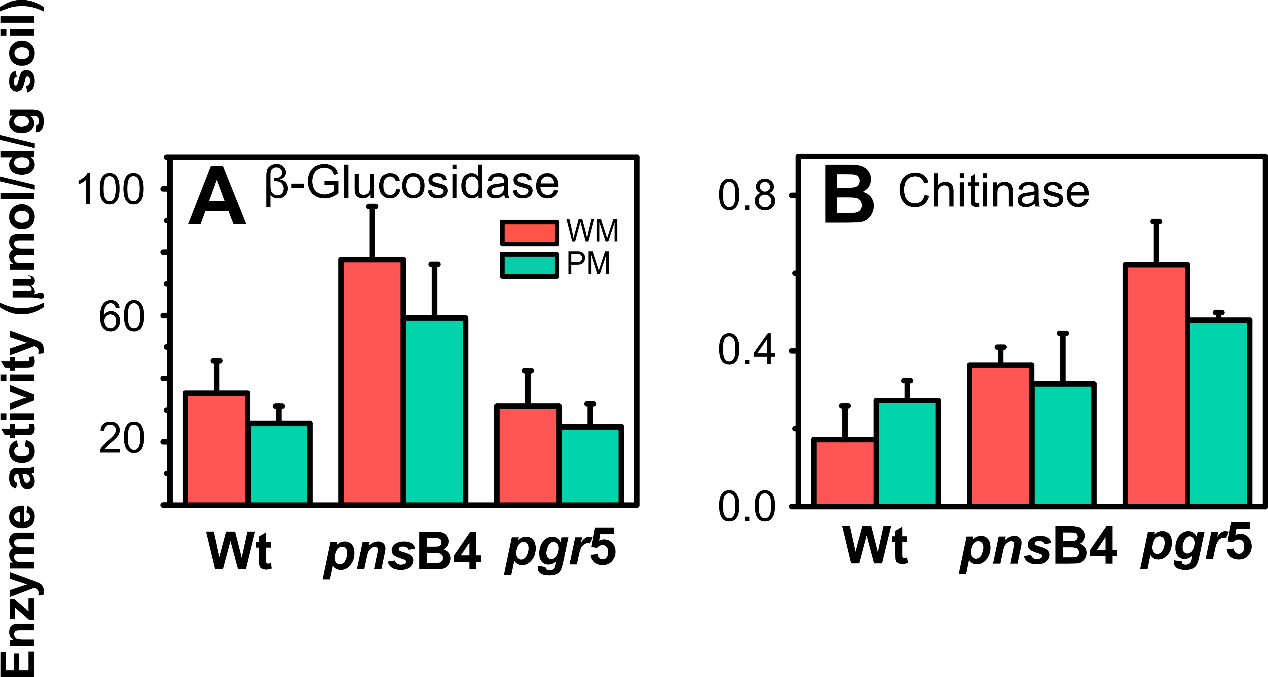


**Fig. S3. Activities of carbon cycle-related enzymes** **in rhizosphere soil.** β-Glucosidase (**A**) and Chitinase (**B**) in rhizosphere soil of the Wt or two mutants (*pns*B4 or *pgr*5) of *Arabidopsis* grown in microcosms in the presence of non-sterilized soil slurry (WM and PM) from three generations. Values are mean ± SD (n= 4).


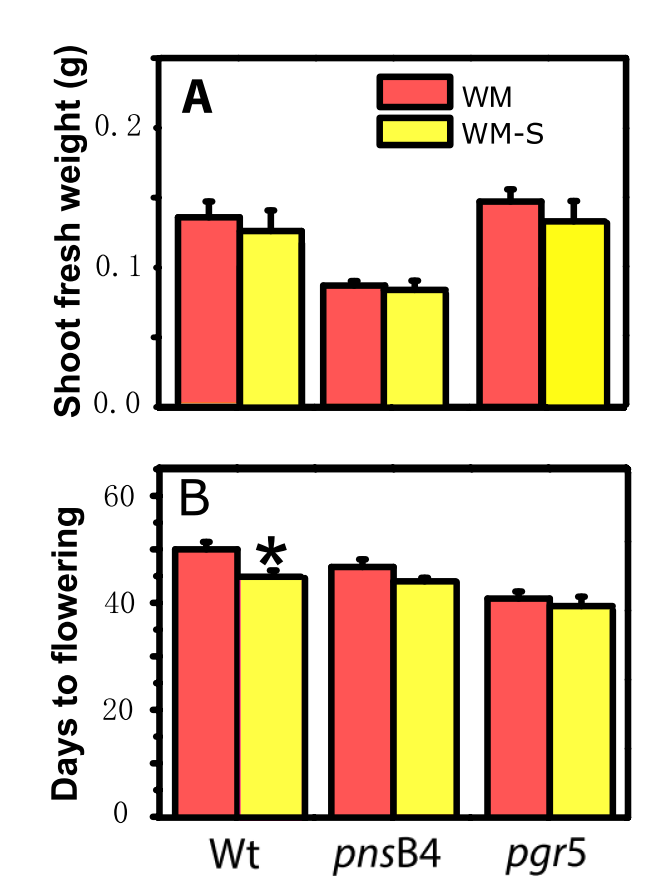


**Fig. S4 Comparisons between WM and WM-S treated plants**. The shoot fresh weight in WM plants tended to be slightly higher than in WM-S plants but there exists no statistically significant difference between the two groups. The number of days to flowering in WM plants was higher than WM-S plants, but this phenomenon only exists in wild-type plants and the two mutants showed no difference. * represents a significant difference between WM and WM-S (*p*<0.05).

**Fig. S5.** **Rarefaction curves of observed species.** There were 18 samples of rhizosphere soils (6 groups, n=3).

**Table S1. Physiological parameters of *Arabidopsis* in three generations.** Physiological parameters were investigated in wild-type (Wt) and *pgr*5 mutant ecotypes grown in microcosms for one to three generations (G1, G2, or G3) after inoculation with different soil microbiomes. Wt-M and *pgr*5-M represent the groups amended with the soil slurry of Wt plants and *pgr*5 mutant plants, respectively. Wt and *pgr*5 represent the corresponding control without addition of microbiomes. Different letters represent significant differences within a generation (ANOVA and LSD; *p*< 0.05). Values are mean ± SD (n = 9, each with 20 *Arabidopsis* seedlings).

|  | Days to flowering | Shoot height (cm) | Number of rosette leaves | Number of siliques |
| --- | --- | --- | --- | --- |
| G1-Wt | 44.25 ± 0.85^a^ | 9.83 ± 0.66^a^ | 12.34 ± 0.78^a^ | 8.25 ± 0.50^a^ |
| G1-Wt-M | 42.75 ± 0.75^a^ | 10.56 ± 0.81^a^ | 12.16 ± 0.34^a^ | 9.00 ± 0.70^a^ |
| G1-*pgr*5 | 38.00 ± 0.82^b^ | 11.72 ± 0.99^a^ | 9.13 ± 0.24^b^ | 7.13 ± 0.98^a^ |
| G1-*pgr*5-M | 38.25 ± 0.63^b^ | 11.35 ± 0.62^a^ | 9.38 ± 0.64^b^ | 7.25 ± 0.67^a^ |
| G2-Wt | 45.33 ± 3.00^a^ | 10.58 ± 0.80^ab^ | 13.48 ± 2.18^a^ | 8.65 ± 2.33^a^ |
| G2-Wt-M | 46.50 ± 1.44^a^ | 9.66 ± 1.22^b^ | 12.37 ± 1.41^a^ | 9.28 ± 3.17^a^ |
| G2-*pgr*5 | 41.00 ± 0.58^b^ | 12.90 ± 0.88^a^ | 9.47 ± 0.34^a^ | 7.29 ± 0.88^a^ |
| G2-*pgr*5-M | 39.75 ± 0.85^b^ | 11.41 ± 0.33^ab^ | 9.32 ± 0.31^a^ | 7.44 ± 0.67^a^ |
| G3-Wt | 40.00 ± 1.00^a^ | 9.54 ± 0.43^a^ | 12.75 ± 0.39^a^ | 8.54 ± 0.77^a^ |
| G3-Wt-M | 43.00 ± 1.07^b^ | 11.14 ± 0.39^ab^ | 13.69 ± 0.81^a^ | 10.08 ± 1.28^b^ |
| G3-*pgr*5 | 37.57 ± 0.67^d^ | 13.20 ± 1.06^b^ | 10.13 ± 0.24^b^ | 7.23 ± 0.47^a^ |
| G3-*pgr*5-M | 33.50 ± 0.89^c^ | 9.17 ± 1.96^a^ | 9.06 ± 0.42^b^ | 4.43 ± 1.14^c^ |

**Table S2. Significant enrichment of rhizosphere microorganisms in the third-generation.** Enriched microorganisms in different ecotypes of *Arabidopsis* were detected based on 16S rRNA gene analyses. Plants were cultivated in microcosms for three generations. The ratio in the microorganism abundance in the rhizosphere of the Wt *Arabidopsis* relative to that measured in the rhizosphere of the *pgr*5 mutant *Arabidopsis* is denoted (Wt/*pgr*5) and the reverse comparison is denoted (*pgr*5/Wt). Mean ratios ≥ 2 or ≤ 0.5 are shown and for which the differences between both *Arabidopsis* ecotypes was significant (ANOVA, n = 3, *p* value < 0.05). N.A.: ratios not available since the abundance of the microorganism genus for either the Wt or the *pgr*5 ecotype was not quantifiable. Detailed taxonomic information was given in Dataset 2.

| Genus | Fold change (Wt/*pgr*5) | Genus | Fold change  (*pgr*5/Wt) |
| --- | --- | --- | --- |
| *Vitellibacter* | 13.00 | *Methyloversatilis* | 16.00 |
| *Coxiella* | 6.33 | *Terrimonas* | 10.13 |
| *Lactobacillus* | 5.00 | *Taibaiella* | 8.67 |
| *Methylotenera* | 4.33 | *Vibrio* | 5.00 |
| *Methylobacterium* | 4.00 | *Emticicia* | 4.50 |
| *Gaiella* | 4.00 | *Legionella* | 3.00 |
| *Bauldia* | 3.50 | *Oligoflexus* | 3.00 |
| *Ferruginibacter* | 3.08 | *Magnetospirillum* | 3.00 |
| *Bacillus* | 2.98 | *Stenotrophomonas* | 3.00 |
| *Salinicola* | 2.76 | *Filimonas* | 3.00 |
| *Starkeya* | 2.70 | *Hydrocarboniphaga* | 2.67 |
| *Arthrobacter* | 2.67 | *Rhodopirellula* | 2.55 |
| *Brevibacillus* | 2.50 | *Pseudospirillum* | 2.50 |
| *Prosthecomicrobium* | 2.50 | *Prosthecobacter* | 2.50 |
| *Singulisphaera* | 2.47 | *Fontimonas* | 2.50 |
| *Alcanivorax* | 2.35 | *Marinicella* | 2.33 |
| *Anaeromyxobacter* | 2.33 | *Asticcacaulis* | 2.14 |
| *Paenibacillus* | 2.28 | *Limnobacter* | 2.13 |
| *Candidatus_Xiphinematobacter* | 2.25 | *SM1A02* | 2.12 |
| *Dyadobacter* | 2.15 | *Panacagrimonas* | 2.10 |
| *Haliea* | 2.12 | *Hydrogenophaga* | 2.06 |
| *Pseudoxanthomonas* | 2.10 | *Nevskia* | 2.00 |
| *Alloprevotella* | 2.00 | *CL500-3* | 2.00 |
| *AKYG587* | N.A. | *Nitrospira* | N.A. |
| *Flavihumibacter* | N.A.. | *Acidibacter* | N.A. |
| *Rhodobacter* | N.A.. | *possible_genus_04* | N.A. |
| *Bacteroides* | N.A. | *BD1-7_clade* | N.A. |
| *Lachnospiraceae_NK4A136_group* | N.A. | *Sorangium* | N.A. |
| *Clostridium_sensu_stricto_1* | N.A. | *Alterococcus* | N.A. |
| *Paucimonas* | N.A. | *Segetibacter* | N.A. |
| *Zavarzinella* | N.A. | *Sulfuritalea* | N.A. |
| *Solirubrobacter* | N.A. | *Runella* | N.A. |
| *Moheibacter* | N.A. | *Altererythrobacter* | N.A. |
| *Rikenellaceae_RC9_gut_group* | N.A. | *Ottowia* | N.A. |
| *Clostridium_sensu_stricto_8* | N.A. | *Candidatus_Odyssella* | N.A. |
| *Nocardioides* | N.A. | *Alkanindiges* | N.A. |
| *Arcicella* | N.A. |  |  |
| *Paracocccus* | N.A. |  |  |
| *Marvinbryantia* | N.A. |  |  |
| *Noviherbaspirillum* | N.A. |  |  |
| *Klebsiella* | N.A. |  |  |

**Table S3. Enriched rare microorganisms in Wt and *pgr*5 *Arabidopsis*.** Rare (relative abundance less than 1%) rhizosphere microorganisms for Wt or *pgr*5 mutant *Arabidopsis* cultures enriched with their respective microbiomes after *Arabidopsis* cultures for three generations in microcosms. Data consistent with 3 replicates. Detailed taxonomic information was given in Dataset 2.

| Enriched in Wt | Enriched in *pgr*5 |
| --- | --- |
| *Arthrobacter** | *Asticcacaulis* |
| *Bacillus** | *CL500-3* |
| *Bauldia* | *Emticicia* |
| *Candidatus_Xiphinematobacter** | *Filimonas* |
| *Gaiella* | *Hydrogenophaga* |
| *Haliea* | *Legionella* |
| *Methylobacterium** | *Marinicella* |
| *Starkeya** | *Panacagrimonas* |
|  | *Rhodopirellula* |
|  | *SM1A02* |
|  | *Stenotrophomonas* |
|  | *Taibaiella* |
|  | *Terrimonas* |

*** microbes (enriched in Wt) that have key roles in rhizospheric N regeneration or in maintaining plant growth

**Table S4.** **Bulk soil properties measured after addition of WM and PM soil slurries.** Bulk-soil pH, available soil K or P contents measured after three generations of Wt *Arabidopsis* cultures in which soil was inoculated with the rhizosphere soil slurry of Wt *Arabidopsis* (Wt-WM) or of *pgr*5 mutant *Arabidopsis* (Wt-PM). Analogously, cultures of the *pns*B4 mutants were inoculated with the soil slurry of Wt *Arabidopsis* (*pns*B4-WM) or of *pgr*5 mutant *Arabidopsis* (*pns*B4-PM). The same notation was used for the *pgr*5 mutant *Arabidopsis* (*pgr*5-WM or *pgr*5-PM). Values are mean ± SD (n = 4). None of the values are significantly different (*p* < 0.05).

|  | pH | Available K content | Available P content |
| --- | --- | --- | --- |
| Wt-WM | 6.32±0.05^a^ | 25.02±0.93^a^ | 95.41 ±1.74^a^ |
| Wt-PM | 6.49±0.05^a^ | 23.26±1.35^a^ | 88.37 ±5.45^a^ |
| *pns*B4-WM | 6.03±0.01^a^ | 24.39± 1.74^a^ | 86.86 ±6.44^a^ |
| *pns*B4-PM | 5.92±0.05^a^ | 23.56± 0.85^a^ | 91.89 ±9.27^a^ |
| *pgr*5-WM | 6.10±0.03^a^ | 23.30±2.53^a^ | 99.44 ±1.01^a^ |
| *pgr*5-PM | 6.02±0.06^a^ | 21.88± 2.74^a^ | 101.45 ±3.72^a^ |

**Table S5. Comparison of root exudates between Wt and *pgr*5 mutant *Arabidopsis*.** Ratio of root exudate concentrations measured in the culture medium of the Wt *Arabidopsis* and those measured in the culture medium of the *pgr*5 mutant *Arabidopsis* after 3 days of hydroponic culture (fold-change or Wt/*pgr5*). Mean ratio for root exudates ≥ 2 or ≤ 0.5 are shown and for which the differences in concentration of a given root exudate between both *Arabidopsis* ecotypes was significant (ANOVA, *p* value < 0.05, n=6).

| Root exudates | Classification | *p* value | Fold change (Wt/*pgr5*) |
| --- | --- | --- | --- |
| Thymine | Nucleoside | 0.0036 | 23.17 |
| Tterephthalic acid | Aromatic carboxylic acid | 0.0249 | 12.30 |
| N, N-dimethyl-L-histidine | Amino acid | 0.0004 | 12.25 |
| M-cresol | Phenols | 0.0026 | 11.07 |
| Benzyl alcohol | Aromatics | 0.0345 | 10.51 |
| 1-Aminocyclopropanecarboxylic acid | Amino acid | 0.0066 | 7.59 |
| Threo-β-hydroxyaspartate | Amino acid | 0.0029 | 6.58 |
| 4-Hydroxy-3-methoxycinnamaldehyde | Phenolic aldehyde | 0.0124 | 5.24 |
| 3, 4-Dihydroxybenzoic acid | Phenolic acid | 0.0235 | 5.16 |
| 1, 5-Anhydroglucitol | Sugar alcohol | 0.0294 | 4.95 |
| N-Acetyl-D-galactosamine | Sugar | 0.0350 | 4.84 |
| Iminodiacetic acid | Carboxylic acid | 0.0020 | 4.60 |
| 5-Hydroxytryptophan | Amino acid | 0.0103 | 4.03 |
| Anabasine | Amide | 0.0043 | 3.32 |
| N-Acetyltryptophan | Amino acid | 0.003 | 2.96 |
| Isoxanthopterin | Pterin | 0.0007 | 2.79 |
| 7, 8-Dimethylalloxazine | Aromatics | 0.0384 | 2.78 |
| cis-Sinapinic acid | Phenolic acid | 0.0066 | 2.34 |
| Methylmalonic acid | Carboxylic acid | 0.0044 | 2.21 |
| 2, 3-Dihydroxypyridine | Pyridine | 0.0072 | 2.09 |
| Phytosphingosine | Fatty acid | 0.0091 | 2.05 |
| Tryptophan | Amino acid | 0.0439 | 2.02 |
| D-alanyl-D-alanine | Amino acid | 3.8E-05 | 0.48 |
| 5-Aminovaleric acid lactam | Amide | 0.0028 | 0.46 |
| Piceatannol | Phenols | 0.0002 | 0.44 |
| 2, 3-Dimethylsuccinic acid | Carboxylic acid | 0.0323 | 0.39 |
| N-Methyl-L-glutamic acid | Amino acid | 0.0023 | 0.34 |
| Biphenyl | Aromatics | 0.0005 | 0.33 |
| Nicotinic acid | Pyridine | 0.0057 | 0.33 |
| Glycine | Amino acid | 0.0035 | 0.32 |
| Vanillylmandelic acid | Aromatic carboxylic acid | 0.0054 | 0.28 |
| Bis(2-hydroxypropyl)amine | Amide | 0.0034 | 0.26 |
| Pentadecanoic acid | Fatty acid | 0.0045 | 0.24 |
| N-α-Acetyl-L-ornithine | Amino acid | 0.0493 | 0.14 |

**Table S6. Sequences of the primer pairs used for qRT-PCR.** Degenerate alphabet, **R**: A or G, **Y**: C or T, **M**: A or C, **K**: G or T, **S**: G or C, **W**: A or T, **B**: G or T or C, **D**: G or A or T, **N**: A or T or G or C.

| Gene | Gene name | Primers | |
| --- | --- | --- | --- |
| 16S | 16S rRNA genes | | Forward:5’- AGAGTTTGATYMTGGCTCAG -3’  Reverse:5’- TTACCGCGGCTGCTGGC -3’ |
| *amo*A | ammonia monooxygenae | | Forward:5’- GGGGTTTCTACTGGTGGT -3’  Reverse:5’- CCCCTCKGSAAAGCCTTCTTC -3’ |
| *nif*H | Nitrogen-fixation | | Forward:5’- CACCMCSATCAATCTGCT -3’  Reverse:5’- GCCATCATTTCGCCGGA -3’ |
| *nir*K | Nitrite reductase | | Forward:5’- ATCATGGTSCTGCCGCG -3’  Reverse:5’- GCCTCGATCAGRTTGTGGTT -3’ |
| *nos*Z | Nitrous oxide reductase | | Forward:5’- CGYTGTTCMTCGACAGCCAG -3’  Reverse:5’- CGCRASGGCAASAAGGTSCG-3’ |
| *cbb*L | Ribulose 1, 5-bisphosphate carboxylase large subunit | | Forward:5’- AAGGAYGACGAGAACATC -3’  Reverse:5’- TGCAGSATCATGTCRTT -3’ |
| β-*glu* | β-Glucosidase | | Forward:5’- TTCYTBGGYRTCAACTACTA -3’  Reverse:5’- CCGTTYTCGGTBAYSWAGA-3’ |
| *chi*A | chitinase A | | Forward:5’- cgtcgacatcgactgggartdbcc -3’  Reverse:5’- acgccggtccagccncknccrta -3’ |
| *PIE*1 | PHOTOPERIOD-INDEPENDENT EARLY FLOWERING1 | | Forward:5’- GTCTGCTTCCGACAGTGCAG -3’  Reverse:5’- TCTCCGTATCTGGCTGCTCA -3’ |
| *FRI* | FRIDIDA | | Forward:5’- TTCTTCTAATGCCTGATCGTGGT -3’  Reverse:5’- CCGCAGCTAATCCTCCTTCA -3’ |
| *VIN*3 | VERNALIZATION INSENSITIVE 3 | | Forward:5’- CGGTGAAGAAGCTCGAAGGT -3’  Reverse:5’- CCATTGCCTGAGAACACAGC -3’ |
| *VRN2* | VERNALIZATION 2 | | Forward:5’- TCTCTGGACGCTGGTGCTAA -3’  Reverse:5’- CCATTGGCTGGACTCTGTGA -3’ |
| *VRN1* | VERNALIZATION 1 | | Forward:5’- AGTGGTTCTGCGACCATCCT -3’  Reverse:5’- AGACATCGAACAGGCCATTG -3’ |
| *FLD* | FLOWERING LOCUS D | | Forward:5’- AGCCACAACAAGGCGATACC -3’  Reverse:5’- GAATGGCACAGGAATGAGCA -3’ |
| *LD* | LUMINIDEPENDENS | | Forward:5’- CGCGTTCAAGGAGGAGATAGA -3’  Reverse:5’- GCAACGACGACATCTTGGAG -3’ |
| *FPA* |  | | Forward:5’- CCGTCTGTTGTGCTCGTTGT-3’  Reverse:5’- AATGGCAACGGCGTAATGTT -3’ |
| *FY* |  | | Forward:5’- ACAGCCTCTTCCAGGTTCCA-3’  Reverse:5’- CCTTGGTGTTGTTGTTGTTGC -3’ |
| *FCA* |  | | Forward:5’- ATGGCACCTCCTGTTGGACT-3’  Reverse:5’- AAGTAGCTGGACGCCAAGGA -3’ |
| *FVE* |  | | Forward:5’- GTGGAGGTGGAACATTGCAG-3’  Reverse:5’- AAGGCTTGGAGGCACAAGTC -3’ |
| *TOC*1 | TIMING OF CAB 1 | | Forward:5’- GTACCAACCTGTTCTCTGACGACAC-3’  Reverse:5’- CCATCAGCACCAAGACCACCATC -3’ |
| *LHY* | LATE ELONGATED HYPOCOTYL | | Forward:5’- AGGTTGTTGTTACTGCCGCTGTG-3’  Reverse:5’- CATCTGGCTGATTCTCATCTGTCTCC -3’ |
| *GI* | GIGANTEA | | Forward:5’-TGAAGTGTCGTCTACCAGCAACAATA3’  Reverse:5’- CCATCAGCACCAAGACCACCATC -3’ |
| *FT* | FLOWERING LOCUS T | | Forward:5’-GAGGTGACTAATGGCTTGGATCTAAGG-3’  Reverse:5’- GGTTGCTAGGACTTGGAACATCTGG -3’ |
| *CO* | CONSTANS | | Forward:5’- GCCATCAGCGAGTTCCAATTCTACC-3’  Reverse:5’- GCATCCTTATCACCTTCTTCACCTTCC -3’ |
| *CCA*1 | CIRCADIAN CLOCK ASSOCIATED1 | | Forward:5’- GCAGAGCCGAGAGCAAGAACAC-3’  Reverse:5’- TCTGTCTCCTGCTCCATCTGAACC -3’ |
| *RGA* | REPRESSOR OF GA1-3 | | Forward:5’- CATAGGAACGACGGTGACGACAAC-3’  Reverse:5’- TTGCGAGTCAACCAGGATAACAGAAC -3’ |
| *GA*1 | GA REQUIRING 1 | | Forward:5’- CCACACTCACTTCGTTCGTCTCG-3’  Reverse:5’- TCACTCTCCGACAATGCTAACTCAAC -3’ |
| *GAI* | GIBB ERELLIC ACID INSENSITIVE | | Forward:5’- TAACCAAGGCGGCGGAGGAG-3’  Reverse:5’- TGCGAGTCAACCAGGACAACATG -3’ |
